# Acute intermittent hemodialysis management: a single center experience

**DOI:** 10.1080/0886022X.2026.2624275

**Published:** 2026-02-11

**Authors:** Yasemin Kıraç, Ayşegül Alpcan, Yaşar Kandur, Hatice Aktaş

**Affiliations:** ^a^ Division of Nephrology, School of Medicine, Kırıkkale University, Turkey; ^b^ Department of Pediatrics, School of Medicine, Kırıkkale University, Kırıkkale, Turkey; ^c^ Division of Pediatric Nephrology, School of Medicine, Kırıkkale University, Kırıkkale, Turkey; ^d^ Department of Internal Medicine, School of Medicine, Kırıkkale University, Kırıkkale, Turkey

**Keywords:** Acute intermittent hemodialysis, acute kidney injury, outcome, creatinine

## Abstract

This retrospective study evaluated prognostic factors affecting outcomes in patients with acute kidney injury (AKI) treated with acute intermittent hemodialysis (IHD). Medical records of 193 patients treated between 2014 and 2024 were reviewed. Patients were categorized as recovered, deceased, chronic kidney disease without dialysis (CKD-ND), or chronic kidney disease with dialysis (CKD-D). The main indications for dialysis were hypervolemia (64.8%) and uremia (24.9%). Overall mortality was 50.8%, while 23.8% recovered, 21.2% developed CKD, and 4.1% became dialysis-dependent. Significant differences among outcome groups were found in age and serum concentration of serum creatinine, urea, sodium, phosphorus, calcium, C-reactive protein (CRP), albumin. Multivariate logistic regression analysis identified lower creatinine and calcium levels, as well as higher sodium, urea, and CRP concentrations, as independent predictors of mortality. In conclusion, our findings highlight the prognostic importance of biochemical and inflammatory markers in AKI patients undergoing IHD. Early identification and correction of electrolyte and inflammatory disturbances may improve patient outcomes.

## Introduction

Acute kidney injury (AKI) refers to a sudden decline in kidney function, which develops over a span of hours to days [1]. Acute kidney injury is most often observed in critically ill individuals and is linked to high rates of morbidity and mortality. Common reasons for initiating renal replacement therapy (RRT) in cases of AKI include uremic syndrome, electrolyte imbalances unresponsive to treatment, severe metabolic acidosis, hypervolemia that doesn’t respond to therapy, and drug toxicity [2,3]. Intermittent hemodialysis (IHD) remains a fundamental method for kidney replacement therapy worldwide, including in high-income countries, to manage patients with severe AKI and those with preexisting kidney issues during critical illness [4,5]. The main purposes of IHD are to remove metabolic waste and to filter out excess fluids. Over recent years, the occurrence of AKI has been rising, with approximately 5% of ICU patients with severe AKI needing RRT [6]. Acute kidney injury in ICU patients often leads to various negative clinical outcomes, including longer ICU stays, poor quality of life, the need for mechanical ventilation, and complications such as acid-base imbalances, fluid overload, hyperkalemia, uremia, weakened immune function, and depression. These factors are strongly connected to high mortality rates for AKI patients, ranging from 40% to 60% [7,8]. This study aims to examine factors that determine the prognosis in patients undergoing dialysis for AKI and emphasize that timely IHD treatment can prevent permanent kidney damage in AKI patients.

## Materials and Methods

The medical records of patients, who were diagnosed with a need for acute dialysis and received intermittent hemodialysis (IHD) treatment in the Nephrology Clinic’s HD unit of our hospital between 2014 and 2024, were retrospectively reviewed. Written informed consent was obtained from all participants prior to enrollment. The patients were followed for at least three months after the onset of AKI and initiation of acute intermittent hemodialysis (IHD), rather than after hospital discharge. The purpose of this follow-up period was to determine renal recovery or progression to chronic kidney disease (CKD) according to the KDIGO criteria. Regarding the exclusion criteria, patients with previously established CKD (known baseline eGFR <60 mL/min/1.73 m^2^ for more than three months), a history of kidney transplantation, or those already receiving any chronic renal replacement therapy (maintenance hemodialysis or peritoneal dialysis) were excluded from the study.

We retrospectively reviewed clinical characteristics, including age and gender; IHD indications, hospitalization days, pre-dialysis blood pressure, biochemical parameters and treatment results. Biochemical parameters were measured from blood samples taken at the initiation of dialysis. Dialysis was stopped when partial renal function recovery occurred, defined as restored diuresis and a decrease in serum creatinine and urea concentrations. After 3 months, some patients were classified as CKD according to the Kidney Disease: Improving Global Outcomes (KDIGO) CKD criteria [9]. The patients were divided into four groups based on their clinical outcomes: recovered, deceased, those with chronic kidney disease who did not require dialysis (CKD-ND), and those with chronic kidney disease who required dialysis (CKD-D). Clinical and pre-dialysis biochemical parameters were compared within these 4 groups. The study protocol was approved by the Kırıkkale University, Faculty of Medicine Hospital Clinical Research Ethics Committee (Date:14.11.2024/Decision no:2024.11.10).

### Statistical analysis

The normality of the data distribution was assessed using the Kolmogorov-Smirnov test. Age, length of hospital stay, creatinine, urea, glucose,CRP (C-reactive protein), Vitamin D, bicarbonate, albumin, uric acid were not normally distributed. In our analysis, the normality of each continuous variable was first assessed using the Kolmogorov–Smirnov test. For variables that showed approximate normal distribution, parametric tests (t-test and ANOVA) were applied. For non-normally distributed data or when assumptions were not fully met, non-parametric alternatives (Mann–Whitney U and Kruskal–Wallis tests) were also performed to confirm the consistency of the results. Regression analysis was used to assess the relationship between paratemeters and the risk of mortality. A *p* value of less than 0.05 was considered statistically significant. Statistical Package for Social Science (SPSS) version 16.0 (SPSS, Chicago, IL, USA) was used for all statistical analyses.

## Results

Of the 193 patients, 57.5% were male. The median age was 76 years (range, 23–99), and the median length of stay was 12 days (range 1–17). Acute kidney injury was diagnosed prior to admission in 76.2% of the patients, and in the hospital in 23.8%. A total of 125 (64.8%) patients underwent dialysis for hypervolemia, 48 (24.9%) for uremia, and the remaining 20 (10.3%) for various other causes (electrolyte imbalances unresponsive to treatment, severe metabolic acidosis, and drug toxicity). Table 1 presents the clinical and biochemical parameters of all patients in the pre-dialysis stage. Overall, these findings highlight the significant biochemical derangements and comorbidities present in patients before initiating dialysis therapy. Of the patients, 50.8% (*n* = 98) died, 23.8% (*n* = 46) recovered, 21.2% (*n* = 41) progressed to chronic kidney disease (CKD), and 4.1% (*n* = 8) developed dialysis-dependent end-stage renal disease.

**Table 1.** Clinical and biochemical parameters at pre-dialysis stage.

	Predialysis
Mean Systolic BP (mmHg)*	125 ± 34
Mean Diastolic BP (mmHg)*	74 ± 18
Median of creatinine (mg/dL)**	3.5 (0.5–14)
Mean Urea (mg/dL)**	148 (13–413)
Mean sodium (meq/L)*	137 ± 10
Mean potassium (meq/L)*	5.0 ± 1.2
Mean calcium (mg/dL)*	8.1 ± 0.9
Mean phosphorus (mg/dL)*	6.0 ± 2.5
Median of glucose (mg/dL)**	132 (37–750)
C-reactive protein (mg/dL)**	78 (1–505)
Mean hemoglobin (g/dL)*	10.9 ± 2.5
Median of Vitamin D (ng/mL)**	8 (2–38)
Median of bicarbonate (mmol/L)**	18 (5–46)
P^H^*	7.30 ± 0.18
Median of albumin (g/dL)**	3 (1.2–5.0)
Median of uric acid (mg/dL)**	8 (2.5–21)

*Mean ± SD, **Median (Min–Max).

The clinical and laboratory parameters of patients in the outcome groups—Deceased (D), Recovered (R), Chronic Kidney Disease without Dialysis (CKD-ND), and Chronic Kidney Disease with Dialysis (CKD-D)—were compared during the pre-dialysis phase. This analysis revealed statistically significant differences across multiple parameters (Table 2).

**Table 2.** Comparison of the outcome groups in mean of clinical and laboratory parameters at pre-dialysis stage.

	Deceased (D)	Recovered (R)	CKD-ND	CKD-D	*p*	hOC
Age (year)**	79 (23–99)	71 (28–95)	72 (52–93)	80 (55–91)	0.019	*p* = 0.002 between D and R; *p* ≤ 0.001 between D and CKD ND
Mean Systolic BP (mmHg)*	123 ± 37	135 ± 34	123 ± 28	103 ± 4	0.315	
Mean Diastolic BP (mmHg)*	73 ± 19	80 ± 18	73 ± 17	63 ± 5	0.251	
Median of creatinine (g/dl)**	2.8(0.7–9)	4.2 (0.5–11.6)	4.4 (1.3–14)	3.9(2.0–11.7)	0.002	*p* = 0.05 between D and R *p* ≤ 0.001) between D and CKD-ND
Mean Urea (mg/dl)**	156 (13–413)	118 (21–323)	160 (59–312)	88 (53–302)	0.036*	*p* = 0.01 between D and R *p* = 0.023 between R and CKD ND
Mean sodium (meq/L)*	139 ± 12	134 ± 9	134 ± 7	135 ± 6	0.003*	*p* = 0.017 between D and Rp = 0.016 between D and CKD-ND
Mean potassium (meq/L)*	5.1 ± 1.2	4.9 ± 1.2	4.8 ± 1.2	4.6 ± 1.2	0.397	
Mean calcium (mg/dl)*	7.8 ± 0.9	8.2 ± 0.7	8.4 ± 0.7	8.1 ± 0.9	0.002	*p* = 0.002 between D and CKD-ND)
Mean phosphorus (mg/dl)*	6.6 ± 2.7	5.3 ± 2.1	5.7 ± 2.2	4.3 ± 2.5	0.005	*p* = 0.03 between D and R
Median of glucose (mg/dl)**	146 (37–750)	134 (62–388)	108 (66–407)	111 (89–247)	0.160	
C-reactive protein (mg/dl)**	132 (0.5–505)	41 (1–388)	57 (1–299)	116 (1–207)	<0.001*	*p* = 0.001 between D and R *p* ≤ 0.001 between D and CKD-ND
Mean hemoglobin (g/dl)*	11.0 ± 2.6	11.3 ± 2.9	10.4 ± 1.7	10.2 ± 1.9	0.298	
Median of Vitamin D (ng/ml)**	9 (3–23)	6.3 (2–37)	9.4 (3–38)	3.4 (3-5.9)	0.190	
Median of bicarbonate (mmol/l)**	17.5 (5–43)	18.9 (6–46.5)	18.8 (10.6–33)	19.9 (15–28)	0.382	
P^H^*	7.27 ± 0.21	7.32 ± 0.16	7.35 ± 0.10	7.35 ± 0.08	0.09	
Median of albumin (g/dl)**	2.6 (1.2–4.6)	3.5 (2–5)	3.2 (1.0–4.3)	3.3 (2.6–4.3)	<0.001*	*p* = 0.001 between D and R *p* = <0.001 between D and CKD-ND p = 0.017 between D and CKD-D
Median of uric acid (mg/dl)**	9.2 (2.8–21)	7.3 (2.5–18)	8.5 (4–15)	6.6 (2.8–10)	0.212	

D: Deceased; R:Recovered.

*Mean ± SD, ** Median (Min-Max).

Age showed a significant difference among groups (*p* = 0.019), with deceased patients being older (median 79 years) compared to recovered (71 years) and CKD-ND groups (72 years), with pairwise significance between D and R (*p* = 0.002), and between D and CKD-ND (*p* < 0.001). Serum creatinine concentrations were significantly lower in the deceased group (median 2.8 mg/dl) compared to recovered (4.2 mg/dl) and CKD-ND (4.4 mg/dL), with *p* = 0.002 overall and significant pairwise differences (*p* = 0.05 and *p* < 0.001, respectively). Conversely, urea concentrations were significantly elevated in deceased patients (156 mg/dL) compared to those who recovered (118 mg/dL, *p* = 0.01). Additionally, the recovered group exhibited higher urea concentrations than the CKD-ND group (*p* = 0.023). Serum sodium concentrations were relatively higher in the deceased group compared to both the recovered and CKD-ND groups (139 mEq/L vs. 134 mEq/L for both, *p* = 0.017 and *p* = 0.016, respectively). Phosphorus and calcium concentrations were also significantly altered, with deceased patients having higher phosphorus (6.6 mg/dL, *p* = 0.005) and lower calcium concentrations (7.8 mg/dL, *p* = 0.002) than other groups. C-reactive protein (CRP) was notably elevated in the deceased group (median 132 mg/dL), with significant differences compared to recovered (41 mg/dL, *p* = 0.001) and CKD-ND (57 mg/dL, *p* < 0.001). Additionally, serum albumin was significantly lower in deceased patients (2.6 g/dL) than in all other groups (*p* < 0.001),

In the univariate logistic regression analysis, various biochemical and clinical factors were assessed for their relationship with mortality (Table 3). Among these, advanced age, elevated creatinine, increased sodium concentrations, reduced calcium, elevated phosphate, high CRP, decreased serum pH, and low albumin were all found to be significantly associated with a higher risk of death. Multivariate logistic regression analysis identified lower creatinine and calcium levels, as well as higher sodium, urea, and CRP concentrations, as independent predictors of mortality (Table 4).

**Table 3.** Univariate regression analysis of variables in terms of risk for death.

Variables	Univariate regression analysis
Age	OR = 1.020, 95% CI 1.000–1.041; *p* = 0.049*
Systolic BP	OR = 0.998, 95% CI 0.986–1.009; *p* = 0.682
Diastolic BP	OR = 0.997, 95% CI 0.975–1.018; *p* = 0.757
Creatinine	OR = 0.3300, 95% CI 0.653–0.871; *p* ≤ 0.001*
Urea	OR = 1.003 95% CI 1.000–1.007; *p* = 0.079**
Sodium	OR = 1.061, 95% CI 1.026–1.097; *p* ≤ 0.001*
Potasium	OR = 1.214, 95% CI 0.958–1.538; *p* = 0.109**
Calcium	OR = 0.549, 95% CI 0.388–0.776; *p* = 0.001*
Phosphate	OR = 1.230, 95% CI 1.083–1.397; *p* = 0.001*
Glucose	OR = 1.002, 95% CI 0.999—1.006; *p* = 0.218
C-reactive protein	OR = 0.518, 95% CI 1.004–1.010; *p* = 0.004*
Hemoglobin	OR = 0.834, 95% CI 0.914–1.146; *p* = 0.779
Vitamin D	OR = 0.986, 95% CI 0.919–1.057; *p* = 0.687
Bicarbonate	OR = 0.978, 95% CI 0.934–1.023; *p* = 0.327
P^H^	OR = 0.124, 95% CI 0.021–0.740; ***p* = 0.022**
Albumin	OR = 0.320, 95% CI 0.199–0.513; *p* ≤ 0.001*
Uric acid	OR = 1.098, 95% CI 0.975–1.237; *p* = 0.121**

*p < 0.05, **p < 0.200.

**Table 4.** Multivariate regression analysis of variables in terms of risk for deaths.

Variables	Univariate regression analysis
Age	OR = 1.039, 95% CI 0.984–1.097, *p* = 0.167
Creatinine	OR = 0.421, 95% CI 0.244–0.726; *p* = 0.002*
Urea	OR = 1.018, 95% CI 1.002–1.034; *p* = 0.026*
Sodium	OR = 1.097, 95% CI 1.016–1.186; *p* = 0.019*
Potasium	OR = 1.083, 95% CI ,0.874–3.721; *p* = 0.111
Calcium	OR = 0.323, 95% CI 0.106–0.982; *p* = 0.046*
Phosphate	OR = 1.127, 95% CI 0.782–1.624; ***p* =** 0.523
C-reactive protein	OR = 1.012, 95% CI 1.002–1.022; *p* = 0.016*
P^H^	OR = 0.487, 95% CI 0.002–133.313; *p* = 0.802
Albumin*	OR = 0.682, 95% CI 0.226–2.056; *p* = 0.496
Uric acid	OR = 1.140, 95% CI 0.922–1.411; *p* = 0.227

^*^ *p* < 0.05.

## Discussion

In this single-center study evaluating acute intermittent hemodialysis in 193 hospitalized patients, we found a notably high in-hospital mortality rate of 50.8%, consistent with prior reports describing the substantial burden of AKI requiring RRT in elderly and comorbid populations [4,10]. Our results provide important insights into biochemical predictors of mortality, emphasizing the significance of hypernatremia, elevated urea and creatinine, high CRP, and hypocalcemia as independent risk factors.

The median age of patients was 76 years, with deceased patients significantly older than those who recovered or developed chronic kidney disease without dialysis. This observation may be attributed to age-related physiological decline, diminished renal reserve, and a greater prevalence of comorbid conditions [11,12]. Advanced age is a well-recognized prognostic factor in AKI, with prior studies showing a clear association between increasing age and higher mortality risk, even after adjusting for severity of illness [13]. While our findings are consistent with existing evidence, age did not remain statistically significant in the multivariate analysis, indicating that biochemical imbalances may serve as more powerful independent predictors of patient outcomes.

Notably, the deceased group exhibited significantly lower serum creatinine concentrations compared to survivors—a counterintuitive observation that has also been documented in critically ill individuals. In such cases, reduced creatinine may indicate diminished muscle mass, poor nutritional status, or underlying chronic conditions, all of which are linked to unfavorable outcomes [14,15]. In addition in multivariate analysis, low creatinine emerged as an independent risk factor for mortality, which can be attributed to the so-called “creatinine paradox” [4]. This interpretation is consistent with the findings of Chang et al., who reported in patients with dialysis-requiring AKI that lower predialysis creatinine concentrations were associated with higher mortality [16].

Conversely, urea concentrations were higher in deceased patients and remained an independent predictor of mortality in the multivariate model. Increased urea concentrations signify not only compromised renal excretory function but also heightened protein breakdown—an indicator of severe systemic disease and a hypermetabolic state [17]. Previous studies have similarly identified elevated blood urea nitrogen as a robust prognostic indicator in AKI requiring dialysis [18].

Hypernatremia emerged as a strong independent predictor of mortality, consistent with prior studies linking dysnatremia to increased ICU mortality [19]. Hypernatremia in AKI often reflects underlying dehydration, osmotic diuresis, or excessive sodium administration and is associated with cellular dehydration, altered mental status, and increased susceptibility to infection [20]. Its presence likely reflects both disease severity and iatrogenic factors. More commonly, hypernatraemia contributes to hemodynamic instability through hypovolemia and can worsen renal ischemia by reducing renal blood flow [21].

Hypocalcemia was also found to be an independent predictor of mortality in our patient group. Calcium homeostasis is frequently disturbed in AKI due to impaired conversion of vitamin D to its active form, phosphate retention, and hypoalbuminemia [22]. Hypocalcemia may impair cardiac contractility and vascular tone, aggravating hypotension and shock states [23].

CRP concentrations were markedly elevated in deceased patients and independently associated with mortality. This highlights the impact of systemic inflammation on AKI outcomes, as CRP serves as a nonspecific indicator of infection, tissue damage, and cytokine-driven inflammation [24]. Elevated CRP may also indicate sepsis-associated AKI, a particularly poor prognosis [25].

Hypoalbuminemia was significantly more common in non-survivors in univariate analysis, though it lost significance in the multivariate model. As a negative acute-phase reactant, low albumin concentration may indicate systemic inflammation and nutritional deficiency. Previous studies have identified hypoalbuminemia as a predictor of mortality in AKI and dialysis patients [26,27], suggesting its role as a general marker of frailty and disease severity.

These findings advocate for the integration of these laboratory markers into prognostic tools for patients receiving acute intermittent hemodialysis. Considering the elevated mortality risk in elderly patients with AKI, prompt identification and early involvement of nephrology specialists are essential. Studies have shown that timely initiation of RRT in appropriate candidates can improve hemodynamic stability and metabolic control, although the optimal timing remains debated [28].

## Limitations

A major strength of this study is the comprehensive biochemical profiling of all patients prior to dialysis initiation, allowing identification of independent predictors of mortality. However, as a single-center retrospective study, our findings may not be generalizable to all populations. Additionally, we lacked data on long-term outcomes beyond hospital discharge, and residual confounding from unmeasured variables (e.g., comorbidity burden, severity scores) is possible. Clinical factors, including the underlying causes of AKI, cardiac function, and diabetes, were not explored in depth, as the main focus of this study was to assess biochemical indicators associated with mortality rather than to investigate comorbid or etiological determinants. Moreover, as a retrospective single-center study, complete and standardized data on the underlying cause of AKI and comorbidity profiles (e.g., diabetes, cardiac dysfunction) were not uniformly available in all patient records, which could have introduced bias or misclassification if included.

## Conclusion

In summary, we found that in patients undergoing acute intermittent hemodialysis, hypernatremia, low creatinine, high CRP, and hypocalcemia independently predicted mortality. These findings underscore the importance of comprehensive metabolic and inflammatory assessment in AKI patients requiring RRT. Targeting these abnormalities, alongside individualized supportive care, may improve patient outcomes. Incorporating inflammatory and nutritional markers into AKI risk prediction tools may enhance prognostication and guide therapeutic decisions.

## Data Availability

The data supporting the findings of this study are available from the corresponding author upon reasonable request. No publicly accessible dataset was generated or analyzed during the current study.
